# Rapamycin Inhibits the Growth and Collagen Production of Fibroblasts Derived from Human Urethral Scar Tissue

**DOI:** 10.1155/2018/7851327

**Published:** 2018-04-17

**Authors:** Delai Fu, Jian Yin, Shanlong Huang, Hecheng Li, Zhaolun Li, Tie Chong

**Affiliations:** ^1^Department of Urology, The Second Affiliated Hospital of Xi'an Jiaotong University, Xi'an, Shaanxi 710004, China; ^2^Department of Urology, Shaanxi Provincial People's Hospital, Xi'an, Shaanxi 710068, China

## Abstract

Rapamycin can inhibit fibroblast proliferation, collagen accumulation, and urethral stricture in rabbits. Transforming growth factor-beta-1 (TGF-*β*1) signaling, with downstream recruitment of Smad2, is known to promote fibrosis. This* in vitro* study examined the effects of rapamycin on fibroblasts derived from human urethral scar tissue (FHUS) and investigated the possible mechanism with respect to regulation of TGF-*β*1 signaling. FHUS were cultured from urethral scar tissues collected from four patients with urethral stricture. The cells were exposed to different concentrations of rapamycin (0, 10, 20, 40, 80, or 160 ng/ml) for 24 or 48 hours. Cell growth was assessed by the MTT assay. Collagen content was measured based on hydroxyproline levels. The mRNA expressions of Smad2, eIF-4E, and alpha-1 chains of collagen types I and III (Col1*α*1 and Col3*α*1) were determined by semiquantitative reverse-transcription PCR. The protein expressions of Smad2, phospho-Smad2, and eIF-4E were evaluated by western blot. Rapamycin caused a concentration-dependent inhibition of FHUS growth at 24 and 48 hours (*P* < 0.01). Rapamycin decreased total collagen content (*P* < 0.01), collagen content per 10^5^ cells (*P* < 0.05), and mRNA expressions of Col1*α*1 and Col3*α*1 (*P* < 0.05) in a concentration-dependent manner. Rapamycin elicited concentration-dependent reductions in the mRNA (*P* < 0.05) and protein (*P* < 0.01) expressions of Smad2 and eIF-4E. The two highest concentrations of rapamycin also enhanced phospho-Smad2 levels (*P* < 0.01). In conclusion, the present study confirmed that rapamycin may reduce the growth and collagen production of FHUS, possibly through inhibition of TGF-*β*1 signaling.

## 1. Introduction

Urethral stricture is a common problem in men, with a reported prevalence of 229–627 per 100,000 males [1]. Urethral stricture has a negative impact on quality of life and exacts an important economic burden [2]. The etiology of urethral stricture is iatrogenic or idiopathic in around two-thirds of cases, with less common causes including trauma, infection, and chronic inflammation [3, 4]. Surgery is the main treatment method for urethral stricture [5, 6]. The surgical techniques used vary widely from dilation to endoscopic internal urethrotomy and urethroplasty, depending on the length and location of the stricture and the experience of the surgeon [5–8]. Nevertheless, multiple complications can occur even when the most appropriate surgical technique is used, with disease recurrence being the most common complication [9]. The occurrence of postoperative complications can make subsequent disease management more complex, and repeat surgery for disease recurrence is associated with worse outcomes [9]. Thus, the management of urethral stricture remains a surgical challenge.

The pathogenesis of urethral stricture involves the process of fibrosis, characterized by overproliferation of fibroblasts and excessive secretion of extracellular collagen, with a change in the relative abundance of collagen types I and III (COL-I and COL-III) [10, 11]. The transforming growth factor-beta-1 (TGF-*β*1) is believed to play an important role in the fibrosis underlying urethral stricture as it can directly stimulate fibroblasts to increase the secretion of collagen [12, 13] as well as inhibit the growth of epithelial cells. The downstream signaling pathway of TGF-*β*1 involves the Smad family of transcriptional activators, with Smad2 and Smad3 playing important roles in fibrosis [14]. The eukaryotic translation initiation factor 4E (eIF-4E) is a rate-limiting component of the eukaryotic translation apparatus that has been reported to be a translational coactivator of TGF-*β* signaling [15].

Rapamycin is a selective inhibitor of the mammalian target of rapamycin (mTOR). Rapamycin has a powerful inhibitory effect on cell proliferation and has been used in cancer therapy and for immunosuppression after transplantation. Recently, rapamycin has been shown to prevent fibrosis in various fibrotic diseases [16]. Furthermore, our previous study demonstrated that rapamycin could inhibit urethral stricture formation in rabbits and that this effect was associated with reductions in fibroblast proliferation and collagen accumulation [17]. Therefore, the aims of the present study were to determine the effects of rapamycin on fibroblasts derived from human urethral scar tissue (FHUS)* in vitro* and to explore the mechanisms underlying the observed effects.

## 2. Materials and Methods

### 2.1. Ethics Statement

This study was approved by the Medical Ethics Committee of Xi'an Jiaotong University and carried out in strict accordance with the recommendations in the Guide for the Medical Ethics Committee of Xi'an Jiaotong University. According to the guidelines of the Second Affiliated Hospital of Xi'an Jiaotong University, our study met the requirements for exemption from informed consent. Nonetheless, all patients agreed that urethral scar tissue that was excised during their surgical treatment for urethral stricture could be used for medical research.

### 2.2. Cell Culture and Experimental Grouping

FHUS were isolated, cultured, and identified as previously described [18]. Urethral scar tissues from four patients were collected after agreement of the patients. The four patients (dates of surgery, resp.: 2016-1-5, 2016-1-18, 2016-3-17, and 2016-3-18) were treated surgically (excision and primary anastomosis) for bulbous urethral stricture at the Department of Urology, the Second Affiliated Hospital of Xi'an Jiaotong University, China. Briefly, urethral scar tissue was isolated, washed in phosphate-buffered saline (PBS), cut into 1-mm^3^ pieces, and seeded and maintained in tissue culture dishes. FHUS were isolated by trypsinization and cultured by further passage in Dulbecco's Modified Eagle Medium (DMEM; Gibco, USA) containing 10% fetal bovine serum (FBS; Gibco, USA).

Cells at passages 3 to 6 were identified by immunocytochemistry for the fibroblast marker vimentin and used for further study. The cells were allocated to six experimental groups based on the concentration of rapamycin (Sigma, USA) administered: 0 (control group), 10, 20, 40, 80, and 160 ng/ml. All cells were cultured in the presence of FBS. All subsequent experiments were performed at least three independent times in each of the four cell lines.

### 2.3. Immunocytochemistry

FHUS were fixed in 4% paraformaldehyde for 30 min at 25°C. Following fixation, cells were permeabilized with 0.3% (v/v) Triton X-100 in PBS and then blocked with 5% bovine serum albumin. Next, the cells were incubated with the anti-vimentin monoclonal antibody (1 : 100; cat number ab8978; Abcam, Cambridge, UK) at 37°C for 1 h and then incubated with the secondary antibody for 30 min at room temperature (1/1000; Zhongshan Golden Bridge Biotechnology Co., Ltd., Beijing, China). The slides were examined using a Nikon DS-Ri1 Eclipse microscope (Nikon, Tokyo, Japan).

### 2.4. Cell Viability

The cells were incubated with various concentrations of rapamycin for 24 or 48 h in the presence of FBS. At the end of the incubation in rapamycin, the cells were washed with PBS (pH 7.4) and incubated for 4 h in MTT (3-(4,5-dimethylthiazol-2-yl)-2,5-diphenyltetrazolium bromide) solution (Boster Biological Technology Co. Ltd, China). Then, 1 ml of dimethyl sulfoxide (DSMO) was added and the absorbance at 570 nm (A570) was measured using an ultraviolet spectrophotometer (BMG Labtechnologies Pty. Ltd., Australia Pacific Head Office). DMSO alone was used as blank. The rate of inhibition of cell growth for the rapamycin-treated cells was calculated using the following formula:

(1) Cell  growth  inhibition  rate=1−A570  of  rapamycin-treated  cellsA570  of  control  group×100%.

### 2.5. Evaluation of Collagen Expression

Collagen content was measured based on the levels of hydroxyproline, its major component [19]. Cells (3 × 10^5^) were seeded in each plate well and cultured for 24 h in the presence of FBS. The cells were then incubated for 48 h with differing concentrations of rapamycin. At the end of incubation in rapamycin, the supernatant was collected and the hydroxyproline content of the supernatant was determined using a hydroxyproline enzyme-linked immunosorbent assay (ELISA) kit (Jiancheng Bioengineering Institute, China), in accordance with the manufacturer's instructions. The cells in each well were digested by the addition of 0.5 ml of 0.25% trypsin and then counted, allowing collagen production per 10^5^ cells to be calculated.

### 2.6. Western Blot

Western blot was performed to evaluate the protein expressions of Smad2 and eIF-4E as well as the Ser465/467 phosphorylation levels of Smad2. FHUS were cultured in 6-well plates for 24 h in the presence of FBS and then exposed to various concentrations of rapamycin for 48 h. Proteins were extracted using radioimmunoprecipitation assay buffer (Fastagen, China). Samples containing 50 *μ*g of protein were separated on 4–20% gradient gels using sodium dodecyl sulfate-polyacrylamide gel electrophoresis, and the separated proteins were transferred to polyvinylidene difluoride membranes. The membranes were incubated overnight at 4°C with primary antibody against Smad2, phospho-Smad2 (Ser465/467), eIF-4E, or *β*-actin (1 : 500; Cell Signaling Technology, USA). After incubation, the membranes were washed and incubated with the secondary antibody (1 : 1500; ZsBio, China) for 1-2 h at room temperature. The data were analyzed using Image Studio Lite Software (LICOR, Homburg, Germany). *β*-Actin served as loading control.

### 2.7. Semiquantitative Reverse-Transcription Polymerase Chain Reaction (RT-PCR)

RT-PCR was employed to detect the mRNA transcription of Smad2, eIF-4E, Col1*α*1 (which encodes the pro-alpha-1 chain of procollagen type I), and Col3*α*1 (which encodes the pro-alpha-1 chain of procollagen type III). After treatment for 48 h with various concentrations of rapamycin in the presence of FBS, the FHUS were harvested and the RNA was extracted using TransZol UP (Transgen Biotech, China), in accordance with the manufacturer's instructions. First-strand cDNA synthesis was performed with oligo (dT) primers using EasyScript™ RT/RI Enzyme Mix (Transgen Biotech, China). PCR was performed with an iQ5q-PCR instrument (Bio-Rad, USA) using TransTaq™ HiFi PCR SuperMixII (Transgen Biotech, China). The PCR conditions were 94°C for 2 min, 35 cycles at 94°C for 30 s, 55°C for 30 s and 72°C for 40 s, and 72°C for 10 min. The primers used for the PCR reactions are shown in Table 1. Relative quantification of cDNA was carried out using agarose gel electrophoresis. The mRNA levels were normalized to that of *β*-actin, which was used as the internal control.

### 2.8. Statistical Analysis

Statistical analysis was carried out using SPSS 15.0 (SPSS Inc., USA). Normality and homogeneity of variance were evaluated prior to statistical analysis. Normally distributed measurement data with homogeneity of variance were expressed as mean ± standard deviation (SD). Comparisons between multiple subgroups were performed using one-way analysis of variance (ANOVA) with the Student-Newman-Keuls post hoc test. Statistical significance was determined as *P* < 0.05.

## 3. Results

### 3.1. Primary Culture and Identification of FHUS

**Figure 1:**
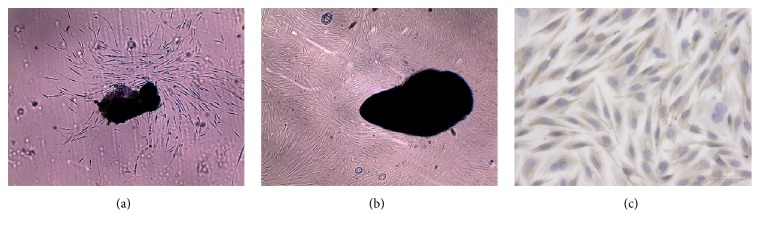
*Primary culture of fibroblasts derived from human urethral scar tissue (FHUS)*. (a) Long, spindle-like cells surrounded the scar tissue after 1 week of tissue culture. Magnification: 40x. (b) A swirl-shaped arrangement of cells was evident after 3 weeks of tissue culture. Magnification: 40x. (c) Immunostaining for vimentin resulted in the staining of filaments within the cytoplasm, indicating that the cells were fibroblasts. Magnification: 400x.

After 7 days of tissue culture, long spindle-like cells were observed around the edge of the urethral scar tissues (Figure 1(a)). These cells proliferated and spread along the wall, to which they adhered. Growth was stopped by contact inhibition. After 3 weeks of culture, the cells covered more than 80% of the view and were arranged in a swirl-shape (Figure 1(b)). Immunocytochemistry for vimentin resulted in the staining of filaments within the cytoplasm, indicating that the cells were fibroblasts (Figure 1(c)).

### 3.2. Rapamycin Inhibits the Growth of FHUS

**Figure 2:**
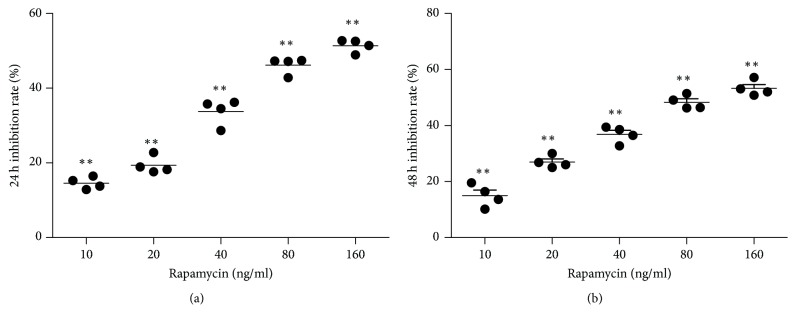
*Rapamycin inhibits the growth of FHUS*. FHUS were exposed to six different concentrations of rapamycin (0, 10, 20, 40, 80, or 160 ng/ml) for either 24 or 48 h. The MTT assay was used to identify viable cells, and the cell growth inhibition rate was calculated as (1 – A570 of rapamycin-treated cells/A570 of control group) × 100%. Rapamycin caused a concentration-dependent inhibition of FHUS growth at 24 (a) and 48 (b) h. Data shown as mean ± standard deviation (*n* = 4 cell lines from four patients).  ^*∗∗*^*P* < 0.01 versus the control group (0 ng/ml rapamycin).

FHUS were exposed to six different concentrations of rapamycin (0, 10, 20, 40, 80, or 160 ng/ml), and the MTT assay was used to determine whether rapamycin inhibited cell growth during culture for 24 or 48 h. Rapamycin caused a concentration-dependent inhibition of FHUS growth at both 24 hours and 48 h (Figure 2), with cell growth significantly reduced at all concentrations of rapamycin (10–160 ng/ml) compared with the control (0 ng/ml) (all *P* < 0.01).

### 3.3. Rapamycin Inhibits Collagen Production by FHUS

**Figure 3:**
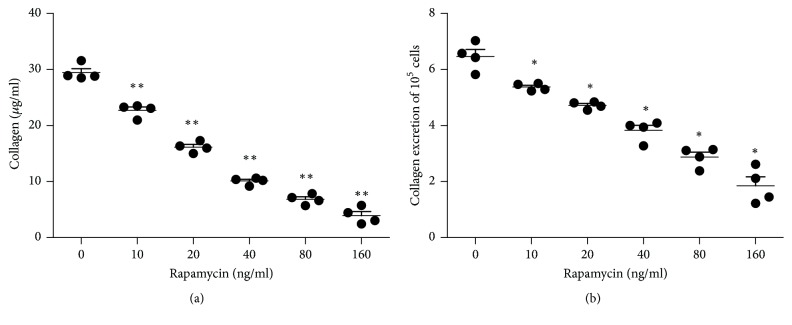
*Rapamycin decreases collagen production by FHUS*. Collagen production by FHUS, based on measurements of hydroxyproline levels (enzyme-linked immunosorbent assay). FHUS were exposed to six different concentrations of rapamycin (0, 10, 20, 40, 80, or 160 ng/ml) for 48 h. (a) Concentration-dependent effects of rapamycin on total collagen production. (b) Concentration-dependent effects of rapamycin on collagen production per 10^5^ cells. Data shown as mean ± standard deviation (*n* = 4 cell lines from four patients).  ^*∗*^*P* < 0.05,  ^*∗∗*^*P* < 0.01 versus the control group (0 ng/ml rapamycin).

**Figure 4:**
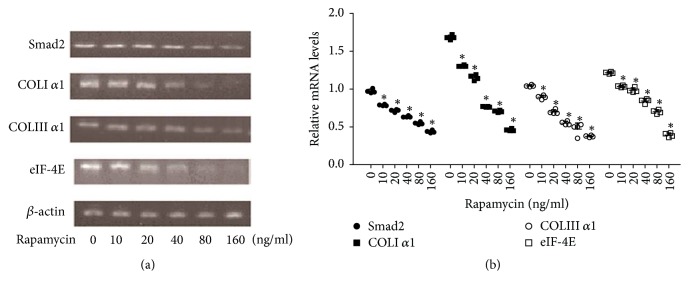
*Rapamycin decreases the mRNA expressions of Smad2, eIF-4E, and the alpha-1 chains of collagen type I and collagen type III (Col1α1 and Col3α1) in FHUS*. The mRNA expressions of Smad2, eIF-4E, Col1*α*1, and Col3*α*1 were determined by semiquantitative RT-PCR using agarose gel electrophoresis, with *β*-actin as internal control. (a) shows representative blots illustrating the effects of various rapamycin concentrations on the mRNA expressions of Smad2, eIF-4E, Col1*α*1, and Col3*α*1. (b) contains quantified data showing the concentration-dependent effects of rapamycin on the mRNA expressions of Smad2, eIF-4E, Col1*α*1, and Col3*α*1. Data shown as mean ± standard deviation (*n* = 4 cell lines from four patients).  ^*∗*^*P* < 0.05 versus the control group (0 ng/ml rapamycin).

Rapamycin (10–160 ng/ml) caused a clear inhibition of collagen production by FHUS (based on measurements of hydroxyproline levels), with concentration-dependent decreases in both total collagen content (*P* < 0.01 versus control for all the other rapamycin concentrations; Figure 3(a)) and collagen content per 10^5^ cells (*P* < 0.05 versus control for all the other rapamycin concentrations; Figure 3(b)). In addition, semiquantitative RT-PCR revealed that rapamycin elicited a concentration-dependent reduction in the mRNA expressions of both Col1*α*1 and Col3*α*1 (*P* < 0.05 versus control for all the other rapamycin concentrations; Figure 4).

### 3.4. Rapamycin Decreases the Expressions of Smad2 and eIF-4E in FHUS

**Figure 5:**
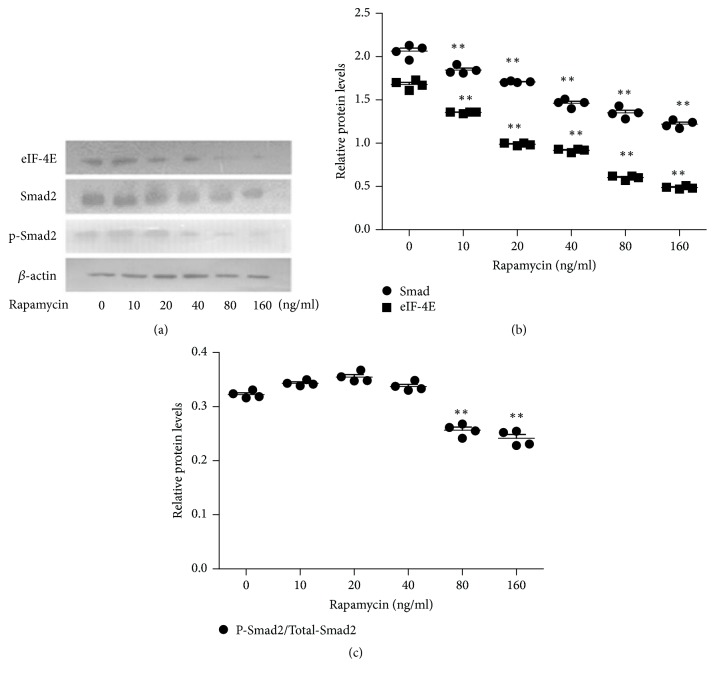
*Rapamycin decreases the protein expressions of Smad2 and eIF-4E and the levels of phosphorylated Smad2 in FHUS*. The protein expressions of Smad2 and eIF-4E and the levels of phospho-Smad2 were measured using western blot, with *β*-actin as internal control. (a) shows representative blots illustrating the effects of various rapamycin concentrations on the levels of eIF-4E, Smad2, and phospho-Smad2 proteins. (b) quantifies the concentration-dependent effects of rapamycin on the protein expressions of Smad2 and eIF-4E. (c) shows the concentration-dependent effects of rapamycin on Smad2 phosphorylation. Data shown as mean ± standard deviation (*n* = 4 cell lines from four patients).  ^*∗∗*^*P* < 0.01 versus the control group (0 ng/ml rapamycin).

Semiquantitative RT-PCR showed that rapamycin reduced the mRNA expressions of Smad2 and eIF-4E in a concentration-dependent manner (*P* < 0.05 versus control for all the other rapamycin concentrations; Figure 4). Western blot demonstrated that rapamycin caused concentration-dependent downregulation of the expressions of Smad2 and eIF-4E proteins (*P* < 0.01 versus control for all the other rapamycin concentrations; Figure 5). At the two highest concentrations (80 and 160 mg/ml), rapamycin also decreased the levels of phospho-Smad2, which indicates an inhibition of TGF-*β*1 signaling.

## 4. Discussion

The main finding of our research was that rapamycin inhibited the growth of FHUS and reduced the expression of collagen by FHUS in a concentration-dependent manner. In addition, rapamycin caused concentration-dependent reductions in the mRNA and protein expressions of Smad2 and eIF-4E, and the two highest concentrations of rapamycin reduced the levels of phospho-Smad2 (i.e., activated Smad2). Since activation of Smad2 is established as a downstream effector of the TGF-*β*1 signaling pathway and since eIF-4E is a translational coactivator of TGF-*β* signaling [15], our data are consistent with the possibility that rapamycin reduces FHUS growth and collagen production, associated with inhibition of TGF-*β*1 signaling.

Our previous study found that rapamycin could inhibit the formation of urethral stricture in rabbits [17]. In the current* in vitro* study, we verified the effects of rapamycin with various concentrations, focusing on FHUS, the primary fibroblasts from human urethral scar tissue. Our results demonstrated that rapamycin significantly inhibited FHUS growth in a concentration-dependent manner, supporting our previous study [17]. This suggests that the inhibition of fibroblasts by rapamycin might not be species-specific. Our finding that rapamycin inhibits the growth of FHUS corroborates the possibility that rapamycin or its analogs could potentially be used clinically as effective inhibitors of human urethral stricture formation.

Collagen is the main component of urethral scars and rapamycin is known to attenuate collagen expression in a variety of fibrotic diseases. Tamaki et al. [19] found that rapamycin could regulate the deposition of COL-I in the extracellular matrix through inhibition of COL-I synthesis and promotion of COL-I degradation. Consistent with this previous investigation, our results indicated that rapamycin evoked concentration-dependent reductions in total collagen production and mRNA expressions of Col1*α*1 (which encodes the pro-alpha-1 chain of procollagen type I) and Col3*α*1 (which encodes the pro-alpha-1 chain of procollagen type III) by FHUS. Additionally, by quantifying collagen production per 10^5^ cells, we demonstrated that the attenuation of collagen production by rapamycin not only was due to inhibition of cell growth (and thus reduced cell numbers compared with the control) but also occurred at the level of individual cells. We hypothesize that rapamycin might decrease cellular metabolic activity without causing cell death, which would be consistent with a recent study of the effects of rapamycin on human foreskin fibroblasts [20].

It is well known that rapamycin binds to mTOR, a key component of mTOR complex-1 (mTORC1), and leads to functional inhibition of mTOR kinase [21]. mTORC1 promotes mRNA translation via phosphorylation of its effectors, ribosomal protein S6 kinase-1 (S6K1), and eIF-4E binding protein-1 (4E-BP1) [22]. Of the two pathways recruited by mTORC1, a recent study indicated that the 4E-BP1/eIF4E pathway may be the most important modulator of collagen expression, with the S6K1 pathway having a minimal effect [23]. eIF-4E, an important component of the translation initiation complex, is normally bound and negatively regulated by 4E-BP1. mTORC1 phosphorylates 4E-BP1, resulting in dissociation of 4E-BP1 from eIF4E, activation of eIF4E, and promotion of translation. The reductions in the mRNA and protein expressions of eIF4E in rapamycin-treated FHUS observed in the present study are consistent with our current understanding of the mTOR signaling pathway.

TGF-*β* is a cytokine widely expressed in fibrotic disorders. During the process of urethral stricture formation, TGF-*β* is thought to play a critical role in the viability and migration of urethral fibroblasts [13] and collagen expression [12]. TGF-*β* signals via types I and II receptors (transmembrane serine/threonine kinases) ultimately phosphorylate receptor-activated Smad2 and Smad3. Crosstalk between the TGF-*β*/Smad and mTOR pathways was recently discovered, with TGF-*β* found to promote collagen production through activation of mTORC1 [24–26]. Our recent study revealed that the expression of TGF-*β* itself was not affected by rapamycin [27]. There is reason to believe that mTORC1 may be an upstream regulator of Smad. Our results provide evidence that rapamycin downregulates Smad2 at both the mRNA and protein levels and reduces Smad2 phosphorylation at Ser465/467. Phosphorylation of Smad2 at Ser465/467 by the activated TGF-*β* receptor is an important component of the TGF-*β*1 signaling pathway [28]. Phosphorylated Smad2 forms a complex with Smad4 that translocates into the nucleus and regulates the transcription of target genes [14]. The phospho-Smad2 level is thus widely used as an indicator of Smad2 activation and TGF-*β* signaling during fibrotic disease [29–31]. A hypothesis consistent with our data is that inhibition of mTORC1 by rapamycin led to attenuation of Smad2 activation by TGF-*β* signaling. Nevertheless, more studies are needed to confirm our findings and test this hypothesis.

## 5. Conclusions

In conclusion, the current findings reveal that rapamycin can inhibit FHUS growth and expression of collagen. The mechanism underlying these effects of rapamycin may involve crosstalk between the mTORC1/4E-BP1 and TGF-*β*/Smad signaling pathways. Rapamycin or its analogs could potentially be used clinically as effective inhibitors of urethral stricture formation. Nevertheless, additional studies are required to confirm and extend our findings and further investigate the relationship between the mTOR and TGF-*β* signaling pathways in urethral stricture formation.

## Figures and Tables

**Table 1:** Primer sequences used for RT-PCR.

Gene	F/R primer	5′ to 3′	Product length
*β*-Actin	F	AGCTACGAGCTGCCTGACG	408 bp
	R	GCATTTGCGGTGGACGAT	
Col1*α*1	F	GTGAGACAGGCGAACAGG	146 bp
	R	GACCAGCAGGACCAGAGG	
Col3*α*3	F	TGCTCCTGGTAAGAAATGG	129 bp
	R	GCCTTGTAATCCTTGTTG	
eIF-4E	F	ATCAAATGCCAAGGGAAACTGGTT	125 bp
	R	TGCAGTGATATCGGACCTAGTTTT	
Smad2	F	TGGGATGGAAGAAGTCAG	95 bp
	R	AAGTGCTTGGTATGGTAAC	

## Supplementary Materials

Supplementary Materials Supplementary materials about the original data associated with Figures 2–5 in this article. Supplementary material for Figure 2: FHUS growth inhibition rate after exposure to rapamycin (0, 10, 20, 40, 80, or 160 ng/ml) for either 24 or 48 h. The cell growth inhibition rate was calculated as (1 – A570 of rapamycin-treated cells/A570 of control group) × 100%. Supplementary material for Figure 3: collagen production by FHUS after treatment of rapamycin (0, 10, 20, 40, 80, or 160 ng/ml) for 48 h based on measurements of hydroxyproline levels (enzyme-linked immunosorbent assay). Supplementary material for Figure 4: the relative mRNA expressions of Smad2, eIF-4E, Col1*α*1, and Col3*α*1 by FHUS after treatment of rapamycin (determined by semiquantitative RT-PCR using agarose gel electrophoresis). Supplementary material for Figure 5: the protein expressions of Smad2, eIF-4E, and ratio of Smad2 phosphorylation to total Smad2 by FHUS after treatment of rapamycin.

## References

[1] Santucci R. A., Joyce G. F., Wise M. (2007). Male urethral stricture disease. *The Journal of Urology*.

[2] Zaid U. B., Hawkins M., Wilson L. (2015). The cost of surveillance after urethroplasty. *Urology*.

[3] Tritschler S., Roosen A., Füllhase C., Stief C. G., Rubben H. (2013). Urethral stricture: etiology, investigation and treatments. *Deutsches Ärzteblatt International*.

[4] Stein D. M., Thum D. J., Barbagli G. (2013). A geographic analysis of male urethral stricture aetiology and location. *BJU International*.

[5] Gallegos M. A., Santucci R. A. (2016). Advances in urethral stricture management. *F1000Research*.

[6] Smith T. G. (2016). Current management of urethral stricture disease. *Indian Journal of Urology*.

[7] Heyns C. F., van der Merwe J., Basson J., van der Merwe A. (2012). Treatment of male urethral strictures—possible reasons for the use of repeated dilatation or internal urethrotomy rather than urethroplasty. *South African Journal of Surgery*.

[8] Burks F. N., Salmon S. A., Smith A. C., Santucci R. A. (2012). Urethroplasty: A geographic disparity in care. *The Journal of Urology*.

[9] Hampson L. A., McAninch J. W., Breyer B. N. (2014). Male urethral strictures and their management. *Nature Reviews Urology*.

[10] Mundy A. R., Andrich D. E. (2011). Urethral strictures. *BJU International*.

[11] Sievert K.-D., Selent-Stier C., Wiedemann J. (2012). Introducing a large animal model to create urethral stricture similar to human stricture disease: A comparative experimental microscopic study. *The Journal of Urology*.

[12] Sangkum P., Gokce A., Tan R. B. W. (2015). Transforming growth factor-*β*1 induced urethral fibrosis in a rat model. *The Journal of Urology*.

[13] Xie H., Feng C., Fu Q., Sa Y.-L., Xu Y.-M. (2014). Crosstalk between TGF-*β*1 and CXCR3 signaling during urethral fibrosis. *Molecular and Cellular Biochemistry*.

[14] Biernacka A., Dobaczewski M., Frangogiannis N. G. (2011). TGF-*β* signaling in fibrosis. *Growth Factors*.

[15] Decarlo L., Mestel C., Barcellos-Hoff M.-H., Schneider R. J. (2015). Eukaryotic translation initiation factor 4E is a feed-forward translational coactivator of transforming growth factor *β* early protransforming events in breast epithelial cells. *Molecular and Cellular Biology*.

[16] Yanaba K. (2016). Strategy for treatment of fibrosis in systemic sclerosis: Present and future. *The Journal of Dermatology*.

[17] Chong T., Fu D.-L., Li H.-C. (2011). Rapamycin inhibits formation of urethral stricture in rabbits. *The Journal of Pharmacology and Experimental Therapeutics*.

[18] Sa Y., Li C., Li H., Guo H. (2015). TIMP-1 induces *α*-smooth muscle actin in fibroblasts to promote urethral scar formation. *Cellular Physiology and Biochemistry*.

[19] Tamaki Z., Asano Y., Kubo M. (2014). Effects of the immunosuppressant rapamycin on the expression of human *α*2(I) collagen and matrix metalloproteinase 1 genes in scleroderma dermal fibroblasts. *Journal of Dermatological Science*.

[20] Gillespie Z. E., Mackay K., Sander M. (2015). Rapamycin reduces fibroblast proliferation without causing quiescence and induces STAT5A/ B-mediated cytokine production. *Nucleus*.

[21] Laplante M., Sabatini D. M. (2012). MTOR signaling in growth control and disease. *Cell*.

[22] Wullschleger S., Loewith R., Hall M. N. (2006). TOR signaling in growth and metabolism. *Cell*.

[23] Walker N. M., Belloli E. A., Stuckey L. (2016). Mechanistic Target of rapamycin complex 1 (mTORC1) and mTORC2 as key signaling intermediates in mesenchymal cell activation. *The Journal of Biological Chemistry*.

[24] Rozen-Zvi B., Hayashida T., Hubchak S. C., Hanna C., Platanias L. C., Schnaper H. W. (2013). TGF-*β*/Smad3 activates mammalian target of rapamycin complex-1 to promote collagen production by increasing HIF-1*α* expression. *American Journal of Physiology-Renal Physiology*.

[25] Das F., Bera A., Ghosh-Choudhury N., Abboud H. E., Kasinath B. S., Choudhury G. G. (2014). TGF*β*-induced deptor suppression recruits mTORC1 and not mTORC2 to enhance collagen i (*α*2) gene expression. *PLoS ONE*.

[26] Das R., Xu S., Nguyen T. T. (2015). Transforming growth factor *β*1-induced apoptosis in podocytes via the extracellular signal-regulated kinase- mammalian target of rapamycin complex 1-nadph oxidase 4 axis. *The Journal of Biological Chemistry*.

[27] Huang S. L., Fu D. L., Li H. C., Zhang P., Chong T. (2016). The effect of rapamycin on TGF*β*1 and MMP1 expression in a rabbit model of urethral stricture. *International Urology and Nephrology*.

[28] Wells R. G., Fibrogenesis V. (2000). TGF-beta signaling pathways. *American Journal of Physiology-Gastrointestinal and Liver Physiology*.

[29] Choi H.-I., Ma S. K., Bae E. H., Lee J., Kim S. W. (2016). Peroxiredoxin 5 protects TGF-*β* induced fibrosis by inhibiting Stat3 activation in rat kidney interstitial fibroblast cells. *PLoS ONE*.

[30] Uemura M., Swenson E. S., Gaça M. D. A., Giordano F. J., Reiss M., Wells R. G. (2005). Smad2 and Smad3 play different roles in rat hepatic stellate cell function and *α*-smooth muscle actin organization. *Molecular Biology of the Cell (MBoC)*.

[31] Liu X., Zhu S., Wang T. (2005). Paclitaxel modulates TGF*β* signaling in scleroderma skin grafts in immunodeficient mice. *PLoS Medicine*.

