# Supplementary material for: Rapamycin Inhibits the Growth and Collagen Production of Fibroblasts Derived from Human Urethral Scar Tissue
Source: Biomed Res Int. 2018 Apr 17;2018:7851327. doi: 10.1155/2018/7851327 (PMC5932518; doi:10.1155/2018/7851327)
Supplement: Supplementary Materials — about the original data associated with Figures 2–5 in this article. Supplementary material for Figure 2: FHUS growth inhibition rate after exposure to rapamycin (0, 10, 20, 40, 80, or 160 ng/ml) for either 24 or 48 h. The cell growth inhibition rate was calculated as (1 – A570 of rapamycin-treated cells/A570 of control group) × 100%. Supplementary material for Figure 3: collagen production by FHUS after treatment of rapamycin (0, 10, 20, 40, 80, or 160 ng/ml) for 48 h based on measurements of hydroxyproline levels (enzyme-linked immunosorbent assay). Supplementary material for Figure 4: the relative mRNA expressions of Smad2, eIF-4E, Col1α1, and Col3α1 by FHUS after treatment of rapamycin (determined by semiquantitative RT-PCR using agarose gel electrophoresis). Supplementary material for Figure 5: the protein expressions of Smad2, eIF-4E, and ratio of Smad2 phosphorylation to total Smad2 by FHUS after treatment of rapamycin. [file 7851327.f1.docx]

**Supplementary materials**

Supplementary materials about the original data associated with Fig2-5 in this article.

**Supplementary material for Figure 2**

FHUS growth inhibition rate after exposeing to rapamycin (0, 10, 20, 40, 80 or 160 ng/ml) for either 24 or 48 h. The cell growth inhibition rate was calculated as: (1 – A570 of rapamycin-treated cells / A570 of control group) × 100%.

| group results | 24h  growth inhibition rate | 48h  growth inhibition rate |
| --- | --- | --- |
| 10 ng/ml | 13.75 | 10.20 |
|  | 12.85 | 16.42 |
|  | 16.49 | 19.54 |
|  | 15.29 | 13.61 |
| 20 ng/ml | 22.78 | 26.85 |
|  | 17.63 | 26.00 |
|  | 18.23 | 30.06 |
|  | 18.94 | 25.04 |
| 40 ng/ml | 36.21 | 38.56 |
|  | 28.68 | 36.51 |
|  | 35.77 | 39.42 |
|  | 34.53 | 32.76 |
| 80 ng/ml | 47.26 | 46.32 |
|  | 47.44 | 51.43 |
|  | 47.16 | 49.12 |
|  | 42.81 | 46.44 |
| 160 ng/ml | 48.91 | 50.85 |
|  | 51.42 | 52.00 |
|  | 52.70 | 57.15 |
|  | 52.56 | 53.09 |

**Supplementary material for Figure 3**

Collagen production by FHUS after treatment of rapamycin (0, 10, 20, 40, 80 or 160 ng/ml) for 48 h. based on measurements of hydroxyproline levels (enzyme-linked immunosorbent assay).

| group results | Collagen production (μg/ml) | Collagen production / 10^5^ cells (μg/ml) |
| --- | --- | --- |
| 0 ng/ml | 28.5 | 5.82 |
|  | 28.83 | 7.03 |
|  | 31.57 | 6.58 |
|  | 28.91 | 6.43 |
| 10 ng/ml | 23.29 | 5.29 |
|  | 20.97 | 5.24 |
|  | 23.53 | 5.47 |
|  | 23.08 | 5.5 |
| 20 ng/ml | 17.31 | 4.81 |
|  | 16.36 | 4.55 |
|  | 15.97 | 4.84 |
|  | 15.02 | 4.69 |
| 40 ng/ml | 10.63 | 3.94 |
|  | 9.19 | 3.28 |
|  | 10.21 | 4.09 |
|  | 10.4 | 4 |
| 80 ng/ml | 7.86 | 3.14 |
|  | 5.72 | 2.38 |
|  | 6.62 | 2.88 |
|  | 7.15 | 3.11 |
| 160 ng/ml | 5.75 | 2.62 |
|  | 4.46 | 2.12 |
|  | 2.44 | 1.22 |
|  | 3.05 | 1.45 |

**Supplementary material for Figure 4**

The ralative mRNA expressions of Smad2, eIF-4E, Col1α1 and Col3α1 by FHUS after treatment of rapamycin (determined by semi-quantitative RT-PCR using agarose gel electrophoresis).

| group results | Smad2 | Col1α1 | Col3α1 | eIF-4E |
| --- | --- | --- | --- | --- |
| 0 ng/ml | 0.96 | 1.68 | 1.03 | 1.21 |
|  | 0.95 | 1.65 | 1.04 | 1.22 |
|  | 1.01 | 1.72 | 1.06 | 1.23 |
|  | 0.97 | 1.68 | 1.04 | 1.2 |
| 10 ng/ml | 0.78 | 1.3 | 0.9 | 1.03 |
|  | 0.8 | 1.3 | 0.89 | 1.05 |
|  | 0.79 | 1.32 | 0.92 | 1.04 |
|  | 0.78 | 1.3 | 0.86 | 1.02 |
| 20 ng/ml | 0.72 | 1.17 | 0.69 | 0.98 |
|  | 0.69 | 1.14 | 0.68 | 0.96 |
|  | 0.72 | 1.19 | 0.74 | 1.03 |
|  | 0.73 | 1.11 | 0.68 | 0.97 |
| 40 ng/ml | 0.63 | 0.77 | 0.56 | 0.85 |
|  | 0.65 | 0.77 | 0.58 | 0.87 |
|  | 0.63 | 0.76 | 0.53 | 0.85 |
|  | 0.63 | 0.76 | 0.53 | 0.8 |
| 80 ng/ml | 0.53 | 0.7 | 0.5 | 0.71 |
|  | 0.57 | 0.71 | 0.53 | 0.73 |
|  | 0.55 | 0.72 | 0.5 | 0.69 |
|  | 0.54 | 0.69 | 0.35 | 0.67 |
| 160 ng/ml | 0.46 | 0.48 | 0.39 | 0.41 |
|  | 0.42 | 0.45 | 0.37 | 0.42 |
|  | 0.44 | 0.45 | 0.36 | 0.36 |
|  | 0.43 | 0.46 | 0.38 | 0.38 |

**Supplementary material for Figure 5**

The protein expressions of Smad2, eIF-4E and ratio of Smad2 phosphorylation to total Smad2 by FHUS after treatment of rapamycin.

| group results | Smad2 WB | eIF-4E WB | p-Smad2/Total Smad2 |
| --- | --- | --- | --- |
| 0 ng/ml | 2.06 | 1.67 | 0.3185 |
|  | 2.1 | 1.7 | 0.3239 |
|  | 2.13 | 1.73 | 0.3309 |
|  | 1.96 | 1.61 | 0.316 |
| 10 ng/ml | 1.81 | 1.34 | 0.3433 |
|  | 1.91 | 1.36 | 0.3498 |
|  | 1.84 | 1.36 | 0.3386 |
|  | 1.82 | 1.36 | 0.3416 |
| 20 ng/ml | 1.71 | 1 | 0.3482 |
|  | 1.7 | 0.97 | 0.3553 |
|  | 1.72 | 1 | 0.3474 |
|  | 1.7 | 0.98 | 0.3674 |
| 40 ng/ml | 1.47 | 0.89 | 0.3301 |
|  | 1.4 | 0.92 | 0.3374 |
|  | 1.47 | 0.93 | 0.3485 |
|  | 1.51 | 0.93 | 0.3331 |
| 80 ng/ml | 1.35 | 0.62 | 0.2616 |
|  | 1.43 | 0.62 | 0.2551 |
|  | 1.28 | 0.6 | 0.2679 |
|  | 1.34 | 0.57 | 0.2415 |
| 160 ng/ml | 1.2 | 0.49 | 0.2545 |
|  | 1.27 | 0.51 | 0.2508 |
|  | 1.17 | 0.47 | 0.2582 |
|  | 1.24 | 0.48 | 0.2522 |
